# Recombinant PrP^Sc^ shares structural features with brain-derived PrP^Sc^: Insights from limited proteolysis

**DOI:** 10.1371/journal.ppat.1006797

**Published:** 2018-01-31

**Authors:** Alejandro M. Sevillano, Natalia Fernández-Borges, Neelam Younas, Fei Wang, Saioa R. Elezgarai, Susana Bravo, Ester Vázquez-Fernández, Isaac Rosa, Hasier Eraña, David Gil, Sonia Veiga, Enric Vidal, Melissa L. Erickson-Beltran, Esteban Guitián, Christopher J. Silva, Romolo Nonno, Jiyan Ma, Joaquín Castilla, Jesús R. Requena

**Affiliations:** 1 CIMUS Biomedical Research Institute and Department of Medical Sciences University of Santiago de Compostela-IDIS, Santiago de Compostela, Spain; 2 CIC bioGUNE, Derio, Spain; 3 Center for Neurodegenerative Science, Van Andel Research Institute, Grand Rapids, Michigan, United States of America; 4 Proteomics Lab, IDIS, Santiago de Compostela, Spain; 5 Centre for Prions and Protein Folding Diseases, University of Alberta, Edmonton, Canada; 6 Priocat Laboratory, Centre de Recerca en Sanitat Animal (CReSA), UAB-IRTA, Barcelona, Catalonia, Spain; 7 USDA, ARS Western Regional Research Center, Albany, California, United States of America; 8 Mass spectrometry Core Facility, RIAIDT, University of Santiago de Compostela, Santiago de Compostela, Spain; 9 Department of Veterinary Public Health and Food Safety, Istituto Superiore di Sanità, Rome, Italy; 10 CIC bioGUNE & IKERBasque, Bizkaia, Spain; Dartmouth Medical School, USA, UNITED STATES

## Abstract

Very solid evidence suggests that the core of full length PrP^Sc^ is a 4-rung β-solenoid, and that individual PrP^Sc^ subunits stack to form amyloid fibers. We recently used limited proteolysis to map the β-strands and connecting loops that make up the PrP^Sc^ solenoid. Using high resolution SDS-PAGE followed by epitope analysis, and mass spectrometry, we identified positions ~116/118, 133–134, 141, 152–153, 162, 169 and 179 (murine numbering) as Proteinase K (PK) cleavage sites in PrP^Sc^. Such sites likely define loops and/or borders of β-strands, helping us to predict the threading of the β-solenoid. We have now extended this approach to recombinant PrP^Sc^ (recPrP^Sc^). The term recPrP^Sc^ refers to *bona fide* recombinant prions prepared by PMCA, exhibiting infectivity with attack rates of ~100%. Limited proteolysis of mouse and bank vole recPrP^Sc^ species yielded N-terminally truncated PK-resistant fragments similar to those seen in brain-derived PrP^Sc^, albeit with varying relative yields. Along with these fragments, doubly N- and C-terminally truncated fragments, in particular ~89/97-152, were detected in some recPrP^Sc^ preparations; similar fragments are characteristic of atypical strains of brain-derived PrP^Sc^. Our results suggest a shared architecture of recPrP^Sc^ and brain PrP^Sc^ prions. The observed differences, in particular the distinct yields of specific PK-resistant fragments, are likely due to differences in threading which result in the specific biochemical characteristics of recPrP^Sc^. Furthermore, recombinant PrP^Sc^ offers exciting opportunities for structural studies unachievable with brain-derived PrP^Sc^.

## Author summary

PrP^Sc^ Prions propagate by inducing the refolding of the natively folded normal cellular prion protein (PrP^C^) into the prion conformation in brain and other mammalian tissues (wild-type). Understanding the structure of PrP^Sc^ is essential to understanding how PrP^Sc^ prions propagate. The secondary structure of PrP^C^ is composed of four α-helical regions, two very small β-sheets and random coil, while PrP^Sc^ is composed entirely of β-sheets and random coil. The β-sheets of PrP^Sc^ wind four times to form a spring-like 4-rung β-solenoid. The rungs are connected by stretches of random coil. We have used proteinase K (PK), to cleave these random coil connecters, allowing us to identify the location of the more PK-resistant β-strand stretches within wild-type PrP^Sc^. In this work, we use recombinant PrP^Sc^, (*in vitro* generated infectious prions) to show that patterns of PK cleavage for recombinant and wild-type PrP^Sc^ are very similar, indicating that they share a common architecture. This also means that recombinant PrP^Sc^ is a true surrogate for wild-type PrP^Sc^. Since recombinant PrP^Sc^ is derived from recombinant PrP, future structural studies employing specific amino acid or stable-isotope labeled amino acid substitutions are easily achievable. Such substitutions are essential for NMR studies.

## Introduction

Prions are infectious proteins [1, 2]. They propagate by inducing the host’s isosequential normal cellular prion protein (PrP^C^) to adopt the infecting prion’s conformation [2]. Prions can be transmitted from one organism to another by different means, for example by oral route [2, 3], hence their infectious nature. Prions pique an extraordinary theoretical and experimental interest because they challenge the notion that only nucleic acids are able to transmit heritable information. But they are also of critical practical importance, since some of them are associated with devastating neurodegenerative diseases. In particular, the mammalian PrP^Sc^ (prion protein, “scrapie” isoform) is the causal agent of the fatal transmissible spongiform encephalopathies (TSEs) [2–4]. TSEs affect both humans and agriculturally important animals, and while PrP^Sc^ prions typically remain contained within a given species, transmission of bovine PrP^Sc^ to humans occurred in the aftermath of the massive European bovine spongiform encephalopathy epizootic, killing more than 200 people and generating widespread alarm [5, 6]. Fortunately, through the intervention of regulators, the crisis has largely abated, although transmission of CJD through blood transfusion remains a concern [5]. This leaves only sporadic Creutzfeldt-Jakob disease (CJD), a rare ailment with a yearly incidence of ~1 case per million people, as the main human prion disease [2, 3].

Elucidating the molecular mechanism that governs the propagation of PrP^Sc^, including the aforementioned transmission barriers has been a central issue and a challenge in prion research since these agents were first discovered [1]. This endeavor has been linked to the quest to elucidate the structure of PrP^Sc^, an obvious pre-requisite to understand how such conformation propagates, *i*.*e*, how it is copied.

In this respect, it is important to note that most known prions, and in particular, PrP^Sc^, form amyloids [4, 7–9]. Therefore, the main force driving and modulating prion propagation must be templating of an incoming partially or totally unfolded prion precursor protein molecule by the upper and lower surfaces of the amyloid fiber. These contain “sticky” β-strands ready to form an array of hydrogen bonds and thereby induce the formation of a new β-strand-rich layer, thus promoting growth of the amyloid filament in the direction of its axis. A recent cryo-electron microscopy study has determined the outline of the architecture of GPI-anchorless PrP^Sc^, showing that it is a 4-rung β-solenoid [10]. This agrees with prior fiber X-ray diffraction [11, 12], 2D electron crystallography [11, 13] and SAXS-based [14] studies of other brain-derived wild type (wt) PrP^Sc^ molecules leading to a similar conclusion. On the other hand, another recent study has shown that shorter PrP sequences, such as PrP23-144, can adopt a flat, in-register amyloid architecture which is also infectious [15].

During propagation of multi-rung β-solenoidal structure, only the upper and lower rungs participate in inter-molecular hydrogen bonding. Identifying the specific amino acid residues that participate in β-strands, in particular those that make up the templating interfaces is essential to understand the details of PrP^Sc^ propagation, and, critically, to understand transmission barriers.

In the past, we have used limited proteolysis to probe PrP^Sc^, in an attempt to identify sequential stretches that comprise β-strands *vs*. those that constitute the random coil loops/turns of PrP^Sc^. It should be noted that, in contrast with early hypotheses, the elegant studies of the Surewicz and Caughey groups [16, 17] demonstrated that no α-helical secondary structure is likely to exist in PrP^Sc^. Data from deuterium/hydrogen exchange studies, and limited proteolysis experiments are incompatible with the presence of any substantial amount of α-helical structure, and a critical reassessment of FTIR studies strongly suggests that absorbance peaks ascribed to α-helices is likely to have been a mis-assignment [9].

Using two analytical approaches, high resolution SDS-PAGE combined with epitope analysis, and mass spectrometry, we identified positions ~116/118, 133–134, 141, 152–153, 162, 169 and 179 (murine numbering) as PK cleavage sites in brain-derived PrP^Sc^. These sites likely define loops and/or borders of β-strands, and are helping us to define the hypothetical threading of the β-solenoid [18].

In this context, recPrP^Sc^ is a very attractive tool for structural studies, given that it allows the introduction of the sequence variations, labels and isotopically labeled amino acid residues necessary for rigorous NMR studies. A number of recombinant PrP preparations with different degrees of infectivity have been described since the seminal report by Legname *et al*.[19–22]. Recently, Wang *et al*. generated recPrP^Sc^ exhibiting incubation times similar to those of brain-derived PrP^Sc^ of the same sequence and causing the same pathogenic changes as that of wt prion disease [23, 24]. While incubation times should be considered very cautiously, given that a long incubation time can be the result of low titer but also of a transmission barrier, the study by Wang *et al*. has led to the definitive acceptance that *bona fide*, highly infectious recPrP^Sc^ can be generated *in vitro*. As a corollary, the possibility to use the versatile recPrP^Sc^ as a convenient model for elucidation of the structure of PrP^Sc^ in general was opened.

Here, we report studies to probe the structure of infectious recPrP^Sc^ using limited proteolysis. Mouse and bank vole (*Myodes glareolus*) recPrP^Sc^ prions yield an array of N-terminally truncated PK-resistant fragments very similar to that seen after PK treatment of brain-derived PrP^Sc^. This is strongly supportive of shared key architectural elements between both prion types.

## Materials and methods

### Reagents and antibodies

The following reagents were obtained from the indicated commercial sources: PNGase F, from New England Biolabs (Ipswich, MA, USA); Tris/Tricine electrophoresis buffer and Broad-Range SDS-PAGE Standards from BioRad (Hercules, CA, USA); Sypro Ruby dye, and Novex Sharp Pre-stained Portein Standard, from Thermo-Fisher (Whaltman, MA, USA); Immobilon P 0,45 μm PVDF membranes, from Millipore (Billerica, MA, USA); Ultra-low Range Molecular Weight Marker, Pefabloc, PMSF and PK, from Sigma-Aldrich (St Louis, MO, USA). All other reagents were from Sigma-Aldrich unless otherwise indicated.

Antibody R1, which recognizes PrP epitope 225–230 [25], was a generous gift from Anna Serban, Institute for Neurodegenerative Diseases, UCSF, and was used at a 1:5000 dilution; antibody #51, which recognizes PrP epitope 92–100 [26] was kindly provided by Lothar Stitz, Fridrich Loeffler Institut, Insel Reims, Germany, and was used undiluted; antibody 3F10, which recognizes PrP epitope 137–151 [27] was a generous gift from Yong-Sun Kim, Hallym University, Republic of Korea, and was used at a 1:5000 dilution. Antibody SAF-84, which recognizes PrP epitope 165–172, was from Thermo Fisher Scientific (Rockford, IL, USA), and as used at a 1:5000 dilution. Secondary antibodies were goat anti-human (Thermo Fisher) and goat anti-mouse (GE Healthcare Life Science, Chicago, IL. USA), used to detect R1, 3F10 and #51, respectively; both were used at a 1:5000 dilution.

### Generation of recPrP

Recombinant mouse PrP23-230 (MoPrP23-230) was expressed in *E*. *coli* competent cells. Bacteria were harvested by centrifugation at 5.000 *g*. Bacterial pellets were lysed by incubation for 30 minutes at room temperature with shaking in lysis buffer (50mM Tris-HCl, 5mM EDTA, 1% Triton X-100, 1mM PMSF, 100 μg/ ml lysozyme, pH = 8); MgCl_2_ and DNase I were then added to 20mM and 5μg/ml final concentrations, respectively, and further incubation at room temperature carried out for 30 minutes. Inclusion bodies thus obtained were collected by centrifugation at 20.000 *g* at 4°C for 20 minutes, and solubilized with inclusion body solubilization solution (6M Gn/HCl, 10 mM of Tris-HCl, 100 mM Na_2_HPO_4_ and 10 mM β-mercaptoethanol, pH = 8.0). The solubilized sample was then filtered through a 0.22 μm filter and loaded to a 5 ml FF Crude His-Trap column (GE Healthcare, Life Sciences (Chicago, IL. USA) connected to a 1200 Series HPLC system (Agilent Technology, Santa Clara, CA, USA). The column was washed with inclusion body solubilization solution and refolded in-column by gradually diminishing the concentration of Gn/HCl and β-mercaptoethanol with a gradient of 10 mM of Tris-HCl, 100 mM Na_2_HPO_4_, pH = 8.0 over 100 minutes; recMoPrP23-230 was then eluted with 30 ml of 200 mM imidazole in10 mM Tris-HCl, 100 mM Na_3_PO_4_,pH = 8.0). The eluate was dialyzed against10mM NaH_2_PO_4_, pH = 5.8, and subsequently against d.i. H_2_O at 4°C. Dialyzed samples were centrifuged to eliminate any aggregated material present and stored at -80°C until used for conversion to recPrP^Sc^.

Bank vole PrP23-231 (BVPrP23-231) was expressed following the same protocol, and similarly applied to a His-Trap column as described above; however, it was eluted from the column by application of a solution consisting of 10 mM Tris-HCl, 2M Gn/HCl, and 100 mM Na_2_HPO_4_, pH = 8.0. Elution fractions containing PrP, as determined by SDS-PAGE with Coomassie staining, were then folded by dialysis against 10 mM sodium acetate, pH = 5. Precipitated material was removed by centrifugation.

### Generation of recPrP^Sc^

Two preparations of recombinant (rec) murine PK-resistant (PK-res) PrP and one of recombinant bank vole PK-res PrP were analyzed in this study; all of them were prepared using different variants of recombinant PMCA (recPMCA). Recombinant MoPrPSc-17kDa was prepared in Grand Rapids (USA) and has been described before [23]. Briefly, it was generated *de novo* from recMoPrP23-230 by recPMCA in the presence of POPG and RNA and its infectivity has been tested in WT mice resulting a 100% attack rate [23, 24].

A recMoPrP^Sc^ (*vide infra* with regard to its infectivity properties) was generated in Bilbao, Spain, from recMoPrP23-230 expressed, purified and folded as described above, by means of recPMCA seeded with recMoPrP^Sc^-17kDa in the presence of 1-palmitoyl-2-oleoyl-sn-glycero-3-phosphoglycerol (POPG) and RNA [23, 24]. This sample was termed recMoPrP^Sc^-950. A sample of recBVPrP^Sc^ (*vide infra*) was prepared by seeded recPMCA using a protocol that will be detailed elsewhere. Briefly, recBVPrP23-231 (109I) prepared, purified and folded as described above, was used as a substrate in a mixture that contains dextran and detergent and that was subjected to several cycles of PMCA; the initial seed used was a small portion of brain homogenate from a bank vole (109I) infected with deer CWD prions.

### Animal studies

#### Ethics statement

Animal experiments were carried out in accordance with the European Union Directive 86/609/EEC on Laboratory Animal Protection. In mouse experiments carried out in Santiago de Compostela procedures and animal care were governed by a protocol that was approved by the Institutional Ethics Committee of the University of Santiago de Compostela (15005/16/006). For the experiments carried out in Rome, bank voles carrying isoleucine at *PRNP* codon 109 [28] were obtained from the breeding colony at the Istituto Superiore di Sanità. The research protocol, approved by the Service for Biotechnology and Animal Welfare of the ISS and authorized by the Italian Ministry of Health (decree number 84/12.B), adhered to the guidelines contained in the Italian Legislative Decree 116/92, which transposed the European Directive 86/609/EEC on Laboratory Animal Protection, and then in the Legislative Decree 26/2014, which transposed the European Directive 2010/63/UE on Laboratory Animal Protection. All efforts were made to minimize animal suffering.

A total of 18 Tga20 mice were intracerebrally (ic) inoculated at 2–3 months of age with 20 μl of recMoPrP^Sc^-950, and5 Tga20 mice were intracerebrally inoculated at the same age with the same amount of recMoPrP^Sc^17kDa. Ten eight-week old bank voles were intracerebrally with 20 μl of a 10^−1^ dilution of PMCA misfolded rec-PrP into the left cerebral hemisphere, under ketamine anaesthesia (ketamine 0.1 μg/g). All animals were individually identified by a passive integrated transponder. The animals were examined twice a week until neurological signs appeared, after which they were examined daily. Diseased animals were humanely euthanized with carbon dioxide at the terminal stage of the disease, but before neurological impairment was such as to compromise their welfare, in particular their ability to drink and feed adequately. Survival time was calculated as the interval between inoculation and euthanasia.

### GPI-less PrP^Sc^

Transgenic homozygous GPI-anchorless (GPI^-^) PrP mice (tg44^-/-^), were obtained by crossing of tg44(^+/-^) heterozygous (GPI^-^) PrP mice [29], generously provided by Bruce Chesebro, Rocky Mountain Laboratories, NIH, USA [26]. This GPI-anchorless tg mouse model is the same that we have used in the past [26]; additional GPI-anchorless tg mouse models have been developed [30]. Female mice were inoculated ic at six weeks of age with 20 μl of a 2% RML-infected mouse brain homogenate, kindly provided by Juan María Torres, CISA, Madrid, Spain. After 365 days post inoculation, mice were euthanized, their brains surgically removed, rinsed in PBS, and stored at -80 °C until needed. A 10% w/v, brain homogenate was prepared in PBS, 5% sarkosyl, using a dounce homogenizer (Wheaton Industries Inc, NJ, USA), followed by one pulse of sonication to clarify the homogenate, with an ultrasonic homogenizer probe (Cole Parmer Instrument CO., Chicago IL, USA). The brain homogenate was treated with 25μg/ml of PK for 30 minutes at 37°C, and then deglycosylated with PNGase F following the manufacturer´s recommendations.

### Limited proteolysis

RecMoPrP^Sc^ was treated with 10 μg/ml PK, at 37°C for 30 minutes. The reaction was quenched by adding 2 mM Pefabloc and incubated for 15 minutes on ice. PK-resistant fragments were then pelleted by centrifugation at 18.000 *g* at 4°C for 1 hour using a (Microfuge 22R centrifuge, Beckman Coulter). Under these conditions, all PK-resistant fragments are recovered in the pellet (S1 Fig). Pellets were resuspended in 6M Gn/HCl and stored at -20°C until use.

### Electrophoresis, western blotting and epitope analysis

PK-resistant fragments were precipitated with ice-cold 85% MeOH. Pellets were resuspended in MiliQ H_2_O and Tricine buffer in a ratio 1:2. Reduction was carried out by adding β-mercatoethanol to 2% (v/v). Samples were boiled for 10 minutes. High resolution electrophoresis was carried as described by Vázquez-Fernández *et al*. [26]. After electrophoresis, gels were washed with miliQ H_2_O and incubated with fixing solution (10% MeOH, 7% acetic acid) for 1 hour at room temperature. Sypro Ruby staining was then performed by incubation overnight at room temperature in the darkness. Alternatively, the gels were transferred to Immobilon P 0.45 μm PVDF membranes, which were subsequently probed with the antibodies described above.

### Mass spectrometry

A 1μL sample of the solution of PK-resistant recPrP^Sc^ fragments solubilized in 6M Gnd/HCl (*vide supra*) was mixed with 49 μL of sinapinic acid solution (10 μg/mL of sinapinic acid dissolved in 30% acetonitrile (ACN) with 0.3% trifluoroacetic acid (TFA) and analyzed by MALDI-TOF. One half μL aliquots were deposited using the dried-droplet method onto a 384 Opti-TOF MALDI plate (Applied Biosystems, Foster City, CA, USA). MALDI analysis was performed in a 4800 MALDI-TOF/TOF analyzer (Applied Biosystems). MS spectra were acquired in linear mode (20 kVsource) with a Nd:YAG, (355 nm) laser, and averaging 500 laser shots. For spectra data analysis of recMoPrP^Sc^ samples, an initial external calibration was carried out using insulin (m/z = 5733), ribonuclease A (m/z = 13682) and lysozyme (m/z = 14305), (Sigma-Aldrich) as standards. A peak with m/z = 9390.2 Da, corresponding to fragment N_153_-S_230_, was unambiguously identified with a mass error < 1 Da by ESI-TOF analysis (*vide infra*) of the same sample (S2 Fig). This peak was used as an internal calibrant, and all m/z values in the spectrum corrected accordingly. For recBVPrP^Sc^ samples, only external calibration was used. The final resulting m/z values were matched to PrP fragments with the help of GPMAW 6.0 software (Lighthouse, Odense, Denmark). Final experimentally calculated mass data are shown in Tables 1 and 2, and match theoretical values within the experimental error of the MALDI-TOF analysis.

**Table 1:** MALDI analysis of PK-resistant fragments in recMoPrP^Sc^-950.

recMoPrP^Sc^-950				
Observed mass (MALDI) (kDa)	Theoretical mass (kDa)	Sequence	Corresponding WB bands	Equivalent PK-resistant fragment(s) in GPI-anchorless PrP^Sc^
13441.1	13431.0	A_116_-S_230_	~14.6 kDa	A_116_/G_118_- S_232_*
11871,3	11867.1	S_134_-S_230_	~13 kDa	M_133_/S_134_—S_232_
11391,9	11395.6	I_138_-S_230_	~12 kDa	G_141_—S_232_
10997.2	11998.1	G_141_-S_230_	~12 kDa	G_141_-S_232_
9514.1	9513.6	N_152_-S_230_	~10.2 kDa	N_152_—S_232_
9399.5^♦^	9399.5	M_153_-S_230_	~10.2 kDa	M_153_-S_232_
8187.9	8184.1	Y_162_-S_230_	~8 kDa	Y_162_-S_232_
6097.7	6103.9	V_179_-S_230_	~6.7 kDa	V_179_-S_232_

*(GPI^-^) PrP mice (tg44^-/-^) express PrP with a sequence that includes two extra serine residues at its C-terminus ([26]).

^♦^Exact value obtained from ESI-TOF analysis (see Materials and methods) and used for internal calibration of MALDI-TOF data.

**Table 2:** MALDI analysis of PK-resistant fragments in recBVPrP^Sc^.

recBVPrP^Sc^				
Observed mass (MALDI) (kDa)	Theoretical mass (kDa)	Sequence	Corresponding Coomassie band number	Equivalent PK-resistant fragment in GPI-anchorless PrP^Sc^
13364	13353.4	A_117_-S_231_	2	A_116_/G_118_- S_232_
11979	11992.3	A_133_-S_231_	3	M_133_/S_134_—S_232_
11789	11790.0	S_135_-S_231_	3	M_133_/S_134_—S_232_
9436	9436.4	N_153_-S_231_	4	N_152_—S_232_

As indicated, 10 μl of PK-resistant recPrP^Sc^ were subjected to ESI-TOF analysis. The sample was injected to an Agilent 1100 HPLC system equipped with a Vydac 218TP C-18 column (Vydac, MD, USA). A gradient of ACN over 0.1% formic acid was applied over 60 minutes, at a flow of 0.2 ml/min. The effluent of the column was fed into a Bruker Microtof Focus mass spectrometer (Bruker Daltonik, Billerica, MA, USA) and sprayed into the mass detector. The capillary voltage was set at 4500 V, the pressure of the nebulizer was 2.5 Bar, the drying gas flow 8 L/minute and the drying temperature, 200 °C. The mass range of the detector was 50–3000 m/z.

## Results

### Generation of recMoPrP^Sc^

**Fig 1:**
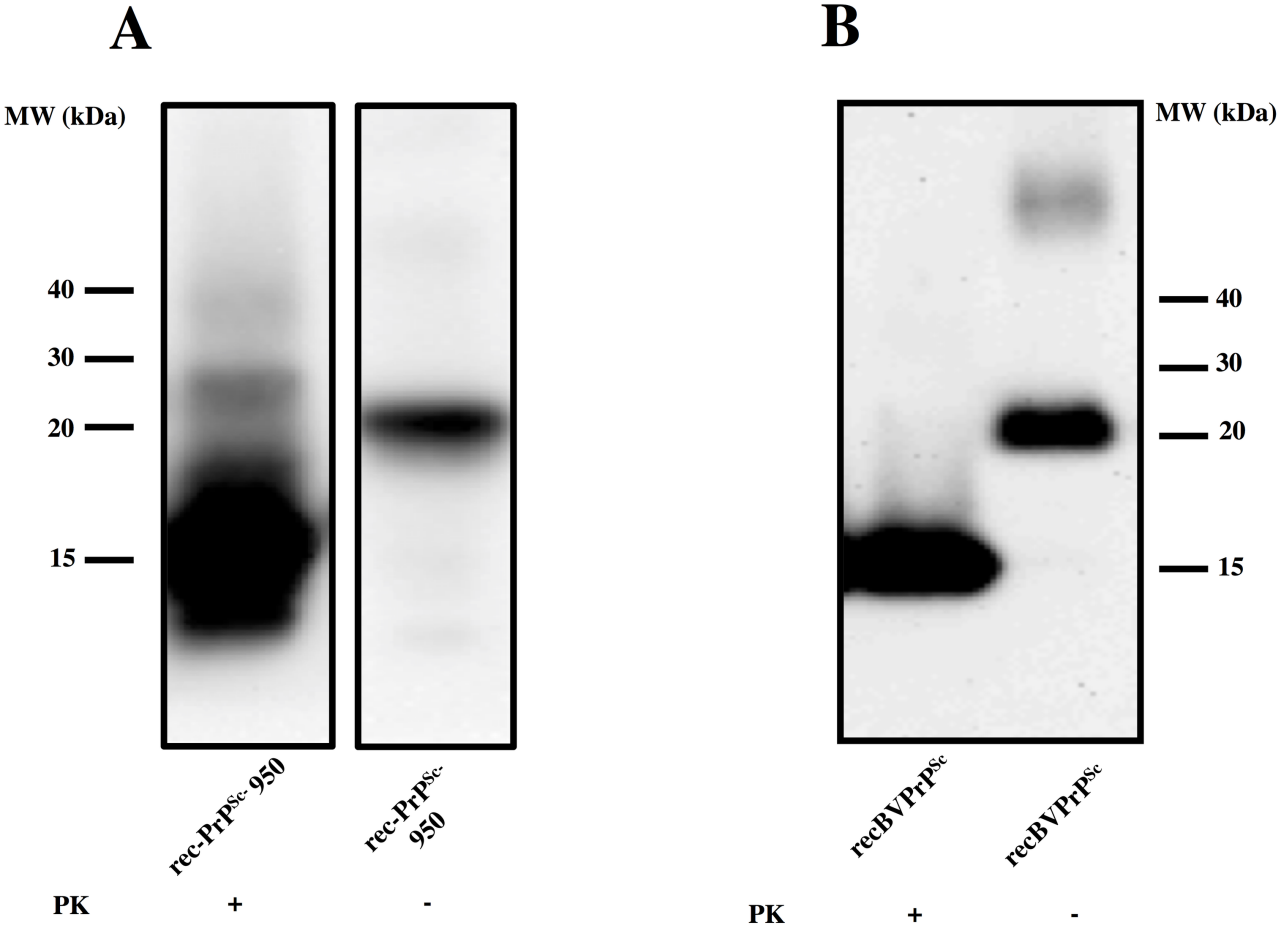
Generation of recPrP^Sc^ *in vitro* using recPMCA: rec-PrP^Sc^ propagate its conformation in vitro. A) A misfolded, propagative mouse PrP species (rec-MoPrP^Sc^-950) was generated in the presence of lipids and RNA[23]using RecMo-PrP^Sc^-17KDa, generated in Grand Rapids (MI, USA), as seed in a recPMCA reaction. The seed was diluted 1:10 in each round of recPMCA up to 17 rounds and the resulting product was digested with 10 μg/ml of PK. The final product is termed “Sc” as its infectivity *in vivo* was later confirmed (Fig 2). B) A misfolded, propagative bank vole PrP species (recBVPrP^Sc^) was generated utilizing a different version of seeded recPMCA (details to be described elsewhere), using recBVPrP23-231 PrP as a substrate. Twenty five rounds were completed. The resulting product was digested with 85 μg/ml of PK. The final sample is termed “Sc” as its infectivity *in vivo* was later confirmed (see Results section). Both samples were analyzed by Western blot using monoclonal antibody Saf83 (1:400). Undigested bank vole and mouse rec-PrP were used as control. MW: molecular weight markers. In A, central lanes with irrelevant samples have been removed from the image for clarity.

**Fig 2:**
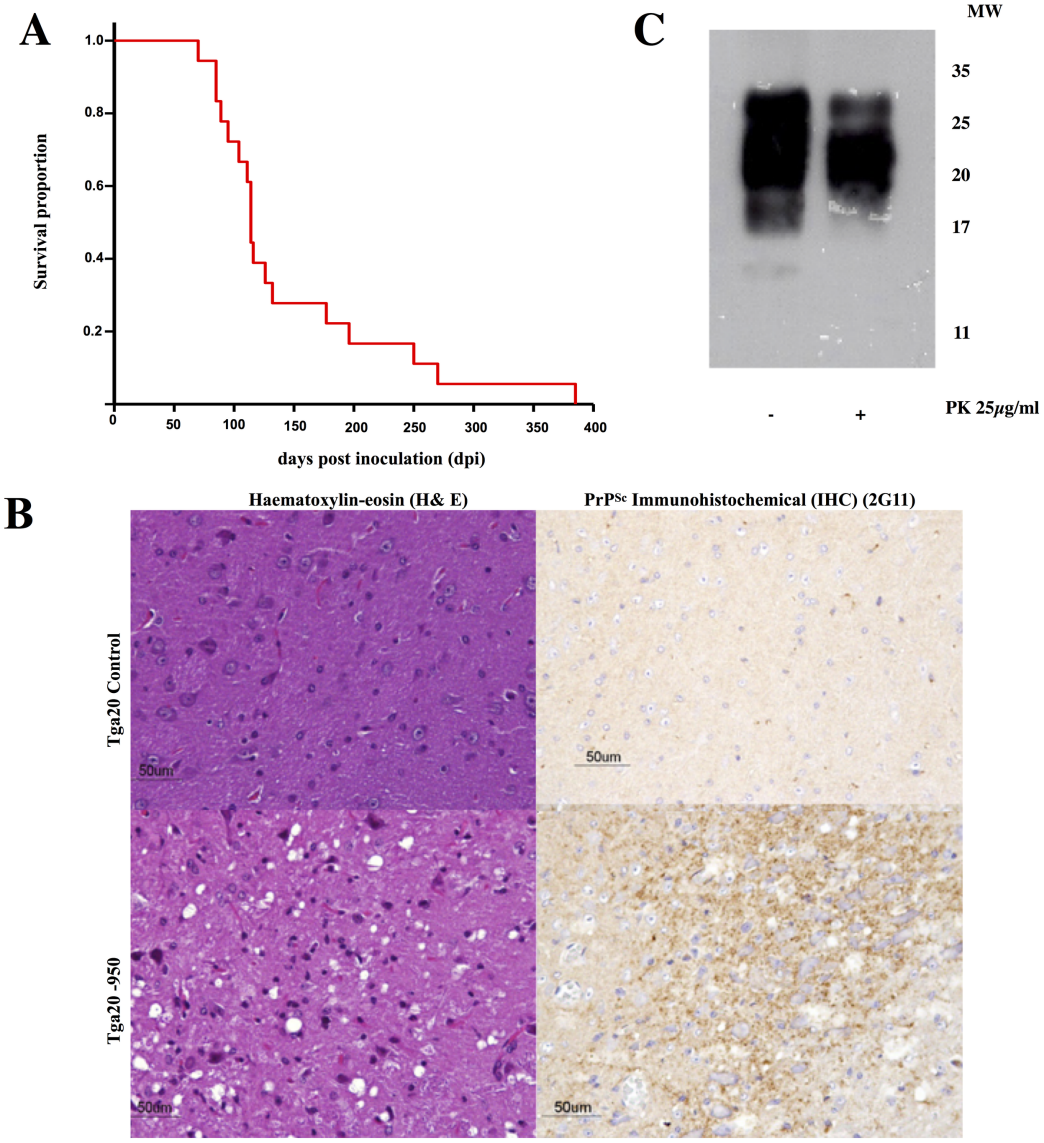
Characterization of the infectivity of recPrP^Sc^. A**)** Kaplan-Meier survival plots of Tga20 mice inoculated with recMoPrP^Sc^. B) Histopathological and immunohistochemical analysis of brains fromTga20 mice inoculated with recMoPrP^Sc^ and uninoculated tga20 controls: left: haematoxylin-eosin (H&E) staining of the medulla oblongata, notice the spongiform lesion in the inoculated mice (bottom); right: PrP^Sc^ IHC staining (antibody 2G11, epitope: 151–159) of the medulla oblongata showing fine granular PrP^Sc^ deposits in the inoculated mice (bottom). C) WB showing the presence of PK-resistant PrP in the brains of Tga20 mice inoculated with recMoPrP^Sc^.

Using seeded recPMCA, we generated a recMoPrP auto-propagative species that we termed recMoPrP^Sc^-950, and was partially resistant to PK (Fig 1A). In order to assess its infectivity, and therefore its prionic nature, we performed animal bioassays. We inoculated this putative recombinant murine prion, into the brains of 18 Tga20 mice. All 18 inoculated Tga20 mice developed standard clinical signs of prion disease (ataxia, hindlimb paralysis, kyphosis, weight loss) and were eventually euthanized (Fig 2A). Histopathological and immunohistochemical examination of the brains of animals inoculated with PK-resistant MoPrP, showed characteristic TSE spongiform lesions and PrP^Sc^ deposits, typical of prion disease (Fig 2B). Furthermore, immunochemical analysis of brain homogenates revealed the presence of PK-resistant PrP (Fig 2C). All this confirms that the inoculated material was a prion, and therefore it could be appropriately referred to as recMoPrP^Sc^-950. In this study we also analyzed a preparation of the recombinant murine prion recMoPrP^Sc^-17kDa [23–24]. Although its infectious nature had already been established [23, 24], we confirmed it under our experimental conditions. We inoculated a group of 5 Tga20 mice, all of which developed signs of prion disease and were humanely euthanized after 119 ± 20 days. Immunohistochemical and immunochemical analysis of brains confirmed prion disease, in agreement with previous published studies [23, 24]

### Structural characterization of recMoPrP^Sc^ using limited proteolysis

**Fig 3:**
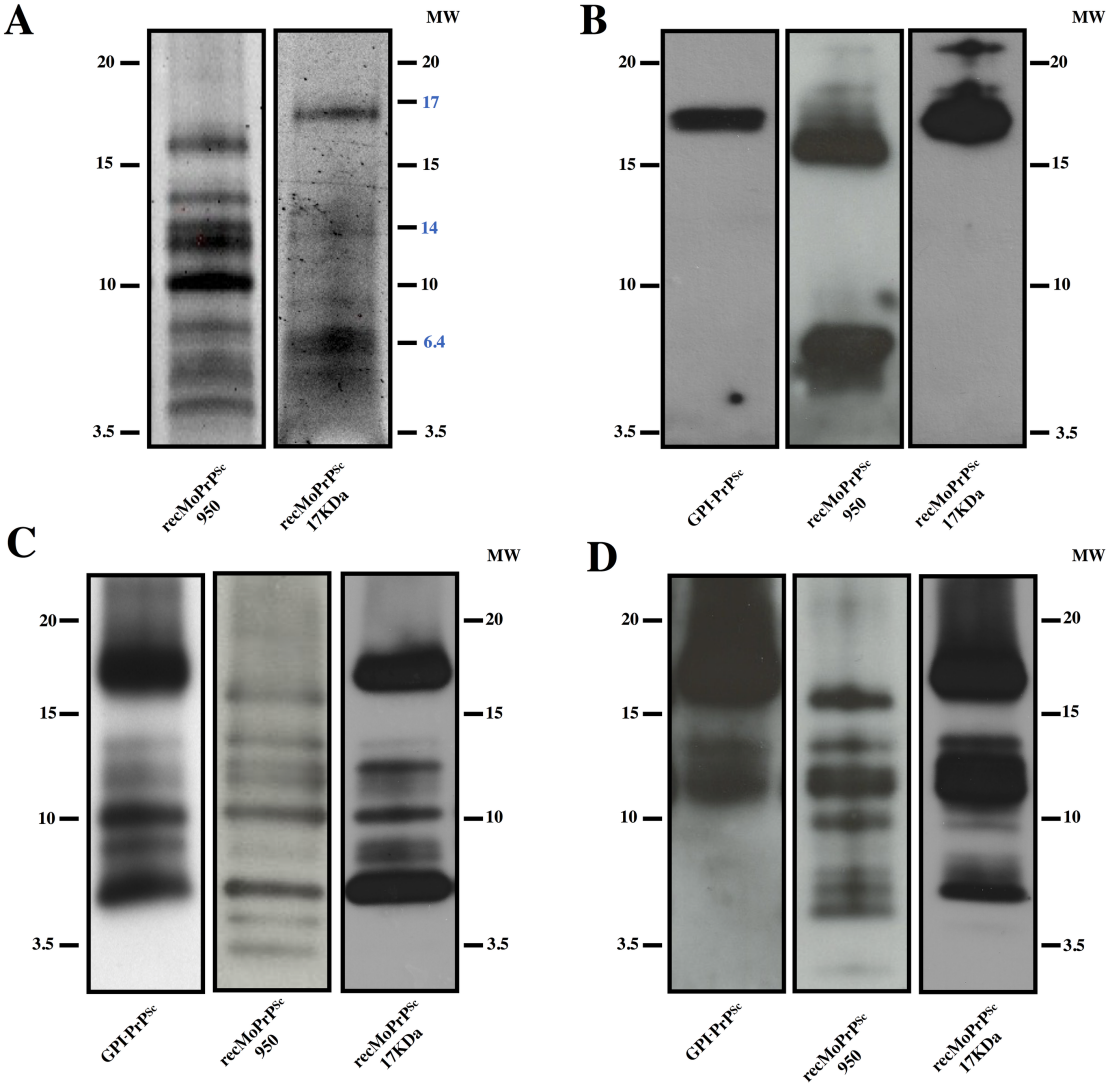
Comparison of PK-resistant fragments from recMoPrP^Sc^ *vs*. brain-derived, GPI-anchorless PrP^Sc^. Recombinant MoPrP^Sc^-950 were treated with PK (see main text for more details)A) Sypro ruby-stained SDS-PAGE gels of recMoPrP^Sc^-950, andrecPrP^Sc^-17kDa. Alternatively, gels were blotted onto PVDF membranes and probed with antibodies: B) #51 (epitope 92–100); C**)** R1 (epitope 225–230); or D) 3F10 (epitope137-151). WBs are representative of several experiments (a minimum of 3 for each antibody).

We subjected recMoPrP^Sc^-950 and recMoPrP^Sc^-17kDa to limited proteolysis using 10 μg/ml of PK for 30 minutes at 37 °C. PK-resistant fragments were detected by Sypro Ruby staining after SDS-PAGE. In recPrP^Sc^-17kDa, a clear, intense ~17 kDa fragment was readily apparent, with two additional somewhat fainter and broader bands, one of ~6.5 kDa, and another with a MW between 6.5 and 3.5 kDa (Fig 3A, left). The ~17 kDa band is obviously equivalent to the classic PrP27-30 PK-resistant fragment seen in brain-derived samples, which migrates between 27–30 kDa [1, 3, 16, 26]. Both PK treated recMoPrP^Sc^-17kDa and PrP27-30 lack amino acids 23–90, so the difference in migration between the two molecules is due to the lack of a GPI anchor and lack of glycosylation in the PK-treated recMoPrP^Sc^-17kDa. This 17kDa eponymous fragment has been previously reported for recMoPrP^Sc^-17kDa [23]. The lower MW PK-resistant fragments may have been previously overlooked, since they would run near the front in conventional SDS-PAGE systems.

In PK treated recMoPrP^Sc^-950 a ~16 kDa band was conspicuous. Additional PK-resistant fragments with smaller apparent MWs between ~15 and ~3.5 kDa, several of them with an intensity similar to that of the uppermost ~16 kDa fragment, were also detected (Fig 3A, right). Compared to the pattern obtained from recPrP^Sc^-17kDa, a number of intense bands in the 15–10 kDa range were seen in recMoPrP^Sc^-950 that were extremely faint or altogether absent in recPrP^Sc^-17kDa (Fig 3A).

We used a set of monoclonal antibodies with well characterized linear PrP epitopes to perform epitope mapping of the PK-resistant fragments present in our samples. We used antibodies recognizing “N-terminal” (antibody #51: 92–100), central (antibody 3F10: 137–151) and C-terminal (antibody R1: 225–230) epitopes. The 92–100 epitope is considered here “N-terminal” given the total absence of any PK-resistant species containing the ~23–90 amino-terminal stretch, believed to be flexible in all PrP conformers. Therefore, position ~90 is considered, for simplicity, to be the reference “amino terminus” of PK-treated samples. The amino acid sequences of mouse and bank vole PrP are shown in S3 Fig to facilitate evaluation of the results.

The use of a high resolution Tris/Tricine SDS-PAGE system optimized the separation of PK-resistant fragments, particularly the smaller ones, while allowing us to compare their pattern with that of PK-treated GPI-anchorless PrP^Sc^, previously analyzed by our group [26]. These results are shown in Fig 3B–3D, which shows results in the following order: “N-terminal”, central and C-terminal antibodies.

The “N-terminal” antibody, #51 (epitope 92–100), detected the ~17 and ~16 kDa bands stained by Sypro ruby in gels corresponding to recPrP^Sc^-17kDa and recMoPrP^Sc^-950, respectively (Fig 3B). In agreement with previous studies [26], antibody #51 recognized the predominant ~17 kDa PK-resistant band in a GPI-anchorless PrP^Sc^-containing sample, which corresponds to (81/89-232) (Fig 3B left); it should be mentioned that GPI-anchorless PrP contains two extra C-terminal amino acid residues as a consequence of the way in which the transgene was designed [26].

Considering their apparent sizes, the ~17 and ~16 kDa PK-resistant fragments in the recPrP^Sc^-17kDa and recMoPrP^Sc^-950 samples (Fig 3B) must correspond to partially overlapping collections of PK-resistant fragments with “ragged termini” [31, 32], with a predominance of cleavages around positions ~86–89 in recPrP^Sc^-17kDa, and 92–98 in recMoPrP^Sc^-950. This difference is reminiscent of the difference between Drowsy *vs*. Hyper or CJD type I *vs*. type II major PK-resistant PrP^Sc^ fragments [31, 32].

The recMoPrP^Sc^-950 sample also contained a broad ~5–7 kDa band, absent in recMoPrPSc-17kDa (Fig 3B center). Considering its apparent MW, it can be concluded that this band necessarily corresponds to doubly N- and C-truncated PK-resistant fragments.

Antibody R1, which recognizes the C-terminal epitope (225–230) detected, as expected, the ~17 and ~16 kDa PK-resistant fragments also seen using Sypro Ruby and antibody #51, in GPI-anchorless PrP^Sc^, recPrP^Sc^-17kDa, and in recMoPrP^Sc^-950, respectively, supporting the notion that these fragments span from ragged ends beginning around position G_92_ all the way to the C-terminus (Fig 3C). In the GPI-anchorless sample, R1 detected, besides, the 6 additional bands previously described and characterized by Vázquez-Fernández *et al*. [26] (Fig 3C left). Remarkably, the pattern of bands detected by this antibody was, to a considerable extent, similar in the other two infectious prion samples, *i*.*e*., recMoPrP^Sc^-17kDa and recMoPrP^Sc^-950. Namely, the ~14.6, ~13, ~12,~10.2, 8, and ~6.7 kDa bands described by Vázquez-Fernández in GPI-anchorless PrP^Sc^ [26] were seen in the GPI-anchorless PrP^Sc^, recMoPrP^Sc^-17kDa and recMoPrP^Sc^-950 samples, although the relative intensities of bands varied from sample to sample (Fig 3C). Considering the extreme C-terminal position of the R1 epitope, which leaves virtually no leeway for alternative sequence combinations leading to a given apparent MW, we can tentatively conclude that there might be a very close identity of these bands between the three samples, which in turn means that cleavage sites are approximately the same. On the other hand, there was one evident difference in the PK digestion pattern of recMoPrP^Sc^-950 with respect to the recMoPrP^Sc^-17kDa and GPI-anchorless samples: two bands, with apparent MWs of ~4.5 and ~3.5 kDa, which are absent in the other samples (Fig 3C). Considering their sizes, they should correspond to novel PK-resistant fragments with N-termini around~G_194_ and ~E_206_, respectively.

The central antibody 3F10 (epitope 137–151) should recognize the ~17/16, ~14.6, ~13 and perhaps the ~12 kDa bands recognized by R1 (Fig 3C), given that they also contain the epitope recognized by 3F10: these bands correspond to fragments ~92/98-230,~116–230, ~134–230 and ~138–230, respectively [26]. Indeed, the antibody revealed bands of these sizes in the two prion samples, albeit with different relative intensities (Fig 3D). Antibody 3F10 also detected additional fragments of ~10, ~8, ~7 and ~6 kDa in the recombinant samples that were not present in GPI-anchorless PrP^Sc^. More specifically, the ~10 kDa band was seen in both samples, while the others were seen in recMoPrP^Sc^-950 (Fig 3D). None of these bands coincides with those recognized by R1 (compare Fig 3D with Fig 3C), whether they have or have not the same size, as there is no possible overlap of epitopes for fragments smaller than 12 kDa. Thus, fragments ~152–230, ~162–230 and ~169/179-230, lack the 3F10 epitope. Therefore, the ~10, ~8, ~7, and ~6 kDa fragments recognized by 3F10 must necessarily correspond to doubly N- and C-terminally truncated PK-resistant fragments. Some of these fragments, more specifically some of those seen in the recMoPrP^Sc^-950 sample, also contain the (92–100) epitope recognized by antibody #51 (Fig 3A), while others do not, and therefore their N-termini must lie beyond the 92–100 sequence. In summary, the combined mapping reveals the existence of a number of PK-resistant fragments with a double truncation at both the N- and C-termini. These fragments are particularly prevalent in recMoPrP^Sc^-950, with N-termini, in this case, around~G_92_. These N-,C-truncated fragments were not seen in PK-treated GPI-anchorless PrP^Sc^ (Fig 3D left), in agreement with previous results [26].

We further probed the PK-resistant fragments in PK-treated recMoPrP^Sc^-950 with an additional antibody, SAF-84 (epitope: 166–172, located between those recognized by 3F10 and R1). Results were consistent with the patterns revealed by these two antibodies and are described in detail in S4 Fig.

**Fig 4:**
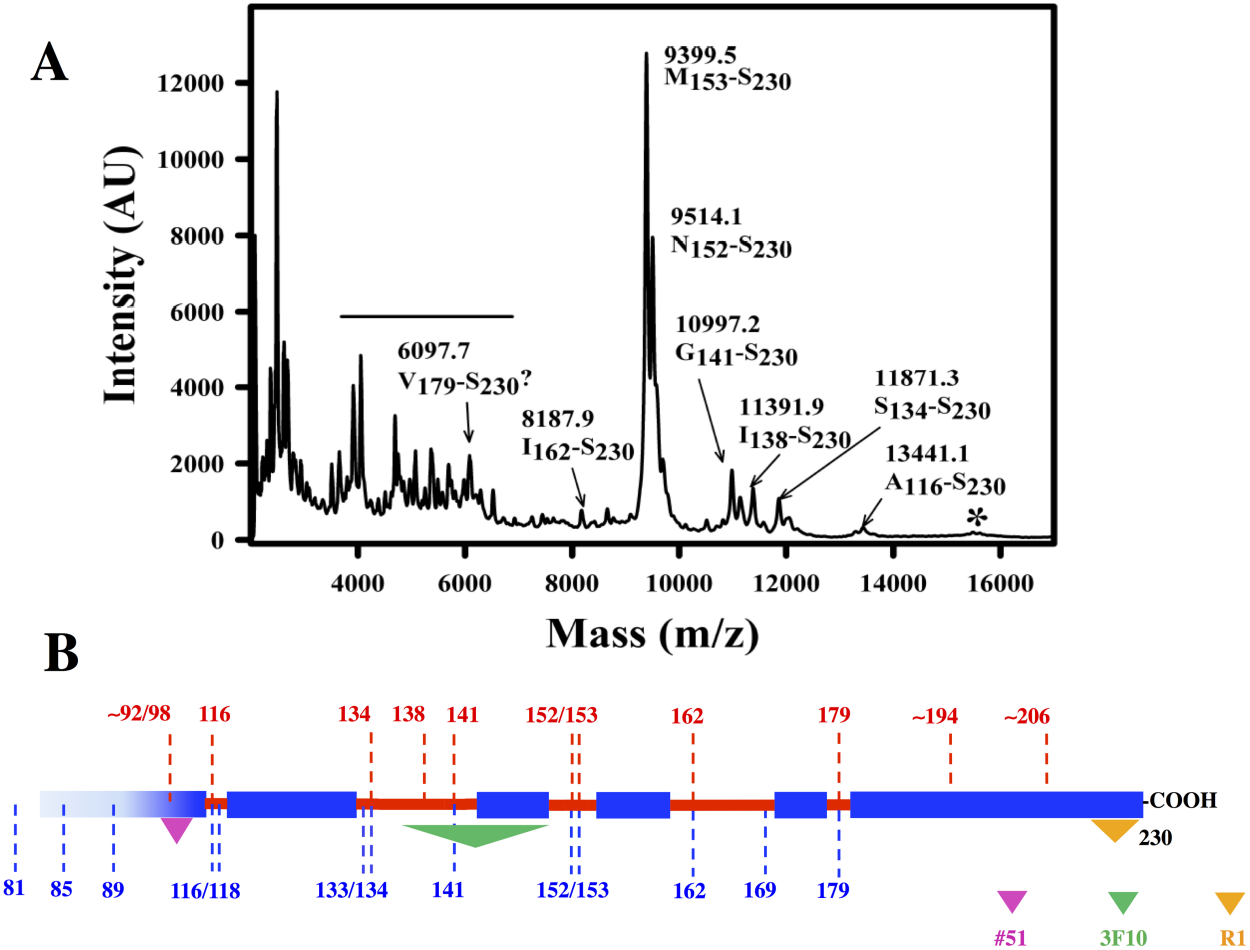
MALDI analysis of PK-resistant fragments from recMoPrP^Sc^. A) MALDI spectrum of recMoPrP^Sc^-950 digested with PK (for more details see the main text). Peak identities were assigned using GPMaw (see text for details). The horizontal line indicates unassigned peaks perhaps corresponding to N-,C- doubly truncated fragments. The asterisk marks poorly defined peaks corresponding to fragments with ragged N-termini beginning at positions ~92–98. B)Scheme showing the location of PK cleavage sites (in red) in the context of the map of PK cleavage sites in GPI-anchorless MoPrP^Sc^ obtained by Vázquez-Fernández *et al*. (in blue, [26]); colored triangles indicate the approximate position of the epitopes of antibodies #51, 3F10 and R1.

We sought to confirm the identity of PK-resistant bands in our recombinant samples, approximately revealed by the sizes and pattern of bands surmised from epitope analysis, by means of mass spectrometry. For logistical reasons, we could only obtain data from recMoPrP^Sc^-950. As shown in Fig 4, Table 1, and S2 Fig, MALDI and ESI-TOF analysis of the same sample identified a number of C-terminal peptides PK-resistant peptides, namely, 89–230, 97–230, 116–230, 134–230, 138–230, 141–230, 152–230, 153–230, 162–230 and 179–230. Such peptides coincide quite well with the apparent MWs of C-terminal peptides detected by antibody R1 (Table 1). Also, in agreement with results obtained with epitope analysis, these peptides are equivalent to those obtained after PK treatment of GPI-anchorless PrP^Sc^ [26].

### Generation and characterization of recBVPrP^Sc^

**Fig 5:**
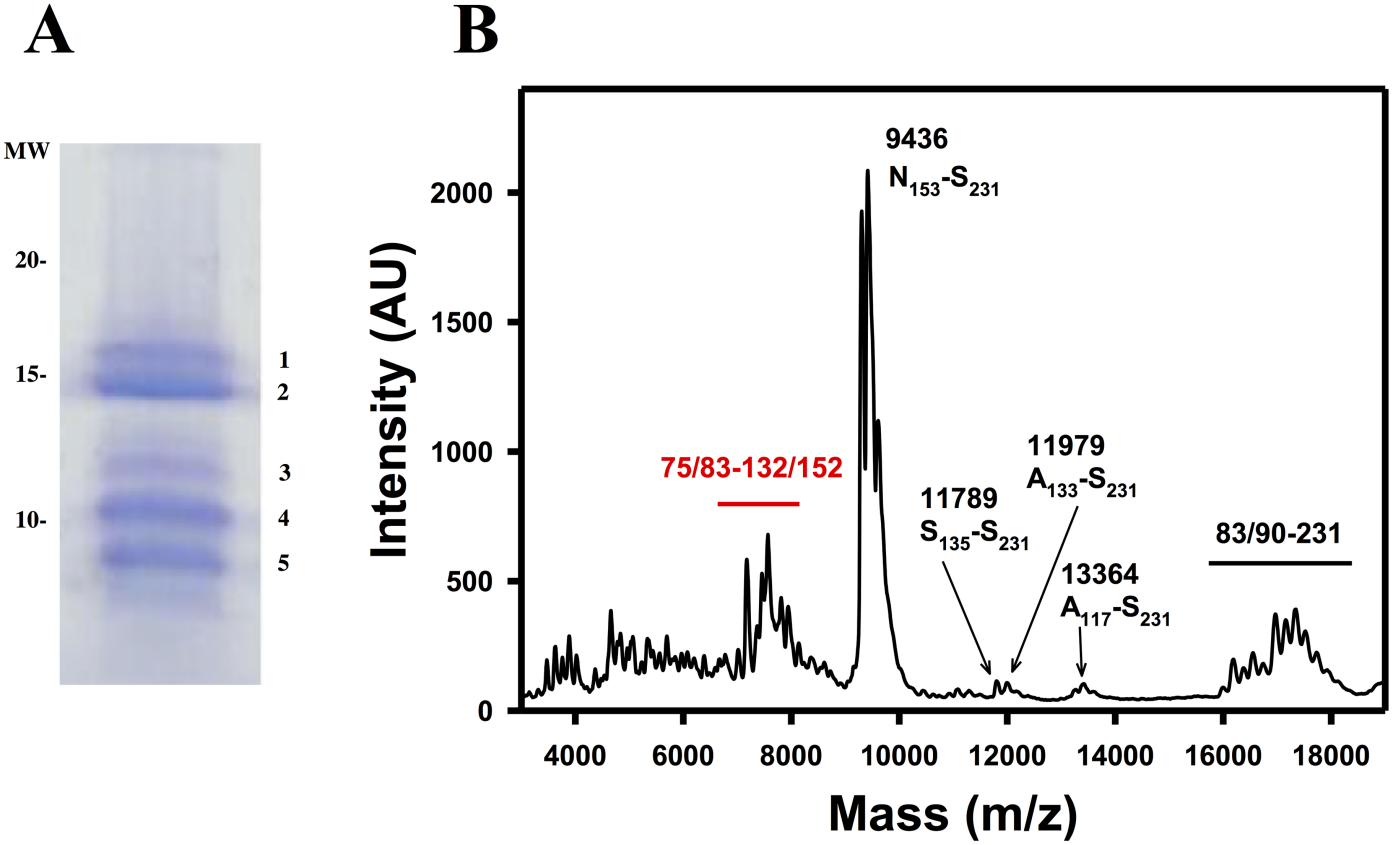
Analysis of PK-resistant fragments of recBVPrP^Sc^. A) Coomassie-stained SDS-PAGE of recBVPrP^Sc^ treated with PK (see text for more details); numbered bands were excised, digested with trypsin in gel and analyzed as described in the Materials and Methods section. B) MALDI spectrum of the same PK-treated sample; the horizontal line in B indicates unassigned peaks perhaps corresponding to N-,C- doubly truncated fragments.

We also generated a recBVPrP auto-propagative species that was partially resistant to PK (Fig 1B). To confirm its infectious, prionic nature, we inoculated this putative recBVPrP^Sc^ into the brains of a group of 10 bank voles expressing homozygous PrP (109I) [33]. Eight of ten bank voles inoculated with putative recBVPrP^Sc^ succumbed to prion disease with a mean survival time of 239 ± 49 days post-inoculation, while the other 2 died of inter-current causes at an early age (206 days post-inoculation), and therefore it cannot be concluded whether they were developing a prion disease or not. Histopathology of brains of these animals confirmed prion disease (S5 Fig). Full details of this prion disease has been reported elsewhere [34]. Therefore, the inoculated material is also a prion and can be appropriately referred to as recBVPrP^Sc^. Treatment of recBVPrP^Sc^ with 20 μg/ml of PK resulted in the appearance of a number of PK-resistant fragments, as seen after Coomassie staining (Fig 5). A doublet of closely migrating ~17 kDa fragments was predominant. We reasoned that it might correspond to the entire PrP sequence minus the extremely flexible ~23–89 tail (Bank vole numbering), with two close but slightly different N-terminal cleavage patterns, *i*.*e*., the equivalent of the ~17 kDa band of GPI-anchorless PrP^Sc^. These two bands were excised and subjected to in-gel tryptic digestion. MALDI analysis of the digest led to the detection of a number of tryptic peptides from amino-terminal (H_111_-R_136_), central (P_137_-R_148_) and carboxy-terminal (E_221_-R_229_) regions of PrP (considering the expected loss of the extreme amino-terminal flexible tail, up to ~G_90_, in fact confirmed by the complete absence of peptides from that region). A characteristic peak with m/z of 1820 Da, corresponding to a “ragged end” tryptic peptide G_90_-K_106_ [35]was also seen in both samples (S6 Fig). No obvious differences in the spectra from the two bands were identified. Since an obvious possibility was the slight difference in apparent MW between the two bands might be the result of different ragged termini, a thorough search for peaks corresponding to tryptic peptides with different ragged termini was carried out, but yielded no obvious differences between the two bands. Therefore, the nature of the difference between the two bands cannot be explained at this point.

In addition to this doublet, at least three additional PK-resistant fragments were detected in the Coomassie-stained SDS-PAGE gel (Fig 5). These lower bands were also subjected to in-gel tryptic digestion followed by MALDI-TOF analysis of the resulting tryptic fragments; MALDI spectra (S6 Fig) showed signals corresponding to tryptic fragments of different regions of the BVPrP sequence, in different proportions, confirming that these bands are PrP fragments of different sizes. In parallel, direct MALDI analysis of the sample confirmed the presence of fragments with sequences 153–231, 135–231, 133–231, 117–231, 75/83-231, and 83/90-231 (Fig 5 and Table 2). A group of peaks corresponding to peptides with masses between ~7000 and ~8000 Da was also evident (Fig 5 and Table 2); these might correspond to doubly N- and C-terminally truncated PK-resistant peptides, similar to those seen in the recMoPrP^Sc^ samples, although this cannot be confirmed at this time.

## Discussion

Recombinant PrP^Sc^ will become an invaluable tool for prion structural studies. Recombinant PrP^Sc^ is produced by converting bacterially derived PrP into recPrP^Sc^, which means that incorporation of stable isotopes or different natural or unnatural amino acids into PrP sequences is greatly simplified. This will allow researchers to prepare custom-made recPrP^Sc^ for NMR-based analyses. However, it will be critical for these efforts to use fully infectious recPrP^Sc^ samples. To date, reports have described studies of amyloid recombinant PrP preparations exhibiting very limited infectivity [36, 37], known to have a structure different to that of PrP^Sc^. On the other hand, very rich structural information is being extracted from highly infectious PrP23-144 amyloid fibers, revealing a flat in-register cross-β stack [15, 38] that is also different from the 4-rung β-solenoid that characterizes full length PrP^Sc^ [10].

Our recPrP^Sc^ prions, made *in vitro* from bacterially derived recombinant PrP, exhibit full infectivity, with attack rates of 100% and incubation periods comparable to wt prions. They share key architectural features with brain-derived PrP^Sc^, when analyzed by a limited proteolysis-based structural analysis [26, 39, 40]. Limited proteolysis of these recPrP^Sc^ species generated a fragmentation pattern consisting of a number of PK-resistant fragments that were the same as or equivalent to those obtained during limited proteolysis of GPI-anchorless MoPrP^Sc^ and wt SHaPrP^Sc^. In particular, mass spectrometry-based analysis revealed nicks at positions 89/90, 116/18, 133/134, 141, 152/153, 162 and 179 (Table 1), in excellent agreement with conclusions derived from epitope analysis (Fig 3).

A group of doubly truncated fragments were much more conspicuous in recMoPrP^Sc^-950 than recMoPrP^Sc^-17kDa. Their exact sequence remains uncertain. However, one of the fragments from recMoPrP^Sc^-950 clearly spans the sequence comprising the epitopes of antibodies #51 and 3F10. This result is consistent with a fragment from positions ~89–152. Such a fragment would complement the fragment spanning the sequence 153–231 see in PK treated recMoPrP^Sc^-950 and GPI-anchorless PrP^Sc^. The theoretical MW of such a fragment would be 6.7 kDa, in good agreement with the band recognized by antibody #51 (Fig 3B). The existence of three distinct bands recognized by 3F10 indicates additional cleavage sites between ~89 and ~152. Based on the analysis of other prions, candidates for these N-terminal cleavage sites are ~117/119 and ~134. Such fragments would be recognized by 3F10 but not #51. For the previously stated reasons, we are unable to define the precise sequences of these peptides. Furthermore, the different responses of different antibodies complicate interpretation of the data. Thus, the ~6.5 kDa fragments detected by 3F10 in recMoPrP^Sc^-17kDa seem not to contain the (92–100) epitope, but in the absence of additional information, it is difficult to conclude where exactly their termini are located.

Doubly truncated fragments have not been associated with the majority of “classical” brain derived prions, such as GPI-anchorless PrP^Sc^ [26], scrapie MoPrP^Sc^, 263K and Dy SHaPrP^Sc^ [39], or CJD PrP^Sc^ [41]. In contrast, low MW bands corresponding to doubly truncated PK-resistant fragments are hallmarks of “atypical” PrP^Sc^ strains, including Gertsmann-Streussler-Scheinker (GSS)-PrP^Sc^, and atypical scrapie-OvPrP^Sc^ strains, such as Nor98 PrP^Sc^ [40, 42]. Thus, analysis of brain homogenates from GSSP102L patients showed the presence of two PK-resistant PrP fragments with apparent molecular masses of ~21 and ~8 kDa. The ~21 kDa fragment, similar to the PrP-res type 1 described in CJD (*i*.*e*., the classic triad of PrP27-30 fragments with variable glycosylation), is typically found in some cases, whereas the ~8 kDa fragment is found in all cases, and has been taken to represent a pathognomonic characteristic of GSS [31, 43]. However, a similar PK-resistant fragment has been also detected in bank vole-adapted CJD PrP^Sc^, blurring to some extent the distinction between classic and atypical strains of PrP^Sc^ [41]. Mass spectrometry-based analysis of the GSS ~8 kDa fragment revealed that it consists of a collection of peptides with ragged termini, spanning from 74/78/80/82 to 147/150/151/152/153 [43]. Furthermore, the ~7 kDa PK-resistant fragment of PrP^Sc^ detected in A117V GSS cases was seen, using mass spectrometry analysis, to span from Gly_88_/Gly_90_ to Arg_148_/Glu_152_/Asn_153_ [44].

As shown by Pirisinu *et al*., this pattern is remarkably similar to that of Nor98 atypical PrP^Sc^, treatment of which with PK yields a ~7 kDa resistant fragment whose sequence is 71/79-153 [40]. In contrast, the most resistant part of PrP^Sc^ from classical strains is, precisely, the complementary sequence: a 152/153-232 fragment becomes prevalent with increasing treatment time with PK of GPI-anchorless PrP^Sc^ [26], and remains folded upon guanidine-induced partial unfolding [26, 45]. All of this suggests that the region around 152/153 marks a “hinge” that connects two stable sub-domains within PrP^Sc^. It is noteworthy that this region signals two halves of the putatively folded region of PrP^Sc^ of comparable size; since the flexible loop likely spans to P_157_ (murine numbering), it would connect two sub-domains of~62 and ~72 residues spanning N- and C-terminally with respect to it.,Higher resistance of either the ~152/153-230 half, typical of “classical” PrP^Sc^ strains [26, 39, 45] (but *vide supra*) or of the ~80/90-152/153 half, characteristic of “atypical strains” [31, 40, 43, 46] might reflect differences in the threading within these specific sub-domains, with differences in the relative content in β-sheet secondary structure (longer or shorter β strands) and packing of the loops connecting them. However, the fact that overall similar nicks are detected in all cases suggests that threading differences are not very large, and that overall, the same elements of secondary structure, likely arranged in the same way, are characteristic of the structures of all three classes of PrP^Sc^. It should also be noted that in any given PrP^Sc^ prion isolate, including our recombinant ones, there might exist mixtures of more than one structure. In that case, the relative abundance of specific PK-resistant fragments will reflect the relative contributions of such structural variants.

Our study also provides preliminary evidence, in recMoPrP^Sc^-950, of two additional, C-terminally located, PK cleavage sites not previously detected in brain-derived PrP^Sc^. The strongest evidence of the existence of such cleavage sites was provided by a ~4.5 kDa band in the Sypro Ruby stained gel of recMoPrP^Sc^ (Fig 3A). A band with a similar apparent MW detected by the C-terminal antibody R1 (epitope 225–230), and since no similar size bands detected by either #51 (epitope 92–100) or 3F10 (epitope 137–151), it follows that there is a PK-resistant fragment spanning from a position around G_194_ to the C-terminus. Furthermore, a band detected by R1 (225–230), with an apparent MW of ~3.5, suggests the existence of a second C-terminal PK-resistant fragment, starting around position E_206_ and spanning to the C-terminus. The absence of a clear equivalent band in the Sypro Ruby-stained gel suggests that the relative abundance of this fragment might be small. Recently, we started to elaborate a generic threading model of PrP^Sc^ by distributing PK-cleavage sites, proline residues and other known structural constraints into a 4-rung solenoid [18]. A cleavage site at G_194_ is compatible with the predicted starting point of the lowermost (C-terminal) rung. However, definitive proof of the identity of these cleavage points should await confirmation by mass spectrometry. Furthermore, given that these cleavage sites have not been detected in GPI-anchorless PrP^Sc^, it remains to be seen whether they are or not a general feature of the architecture of PrP^Sc^.

Ours is not the first structural study of recPrP^Sc^. Recently, Noble *et al*. probed the structure of an infectious recPrP^Sc^ sample by deuterium/hydrogen exchange followed by pepsin digestion and mass spectrometric analysis [47]. They found very substantial protection (*i*.*e*., resistance to exchange) in a stretch spanning from position ~89 up to the C-terminus, suggestive of a β-sheet-rich secondary structure. Short stretches exhibiting somewhat lower protection suggest the presence of loops/turns. In particular, the R_150_-Y_154_ stretch stands out as the possible location of a loop. The furthermost C-terminal stretch Y_224_-S_230_ also shows slightly decreased protection. These results are very similar to those reported by Smirnovas *et al*. in a similar analysis of GPI-anchorless PrP^Sc^ [17]. These authors found substantial protection, indicative of compact, β-sheet-rich structure, from G_81_ up to the C-terminus, with a lower protection from Y_224_ to the C-terminus. These results support the notion that the structure of the recPrP^Sc^ prepared by Noble *et al*. is similar to that of GPI-anchorless PrP^Sc^, in agreement with the results reported here. It should be noted that the pattern of exchange of a non-infectious recPrP amyloid sample was very different, with low exchange rates seen only beyond position ~160 [17].

In summary, our studies show that several infectious mouse and bank vole recPrP^Sc^, generated with the concourse of PMCA, exhibit biochemical properties that strongly suggest that they share key architectural elements with brain-derived PrP^Sc^. Furthermore at least in the case of the mouse sample that we have obtained and studied, they seem to feature a mixture of structural properties of “classical” and “atypical” strains of brain PrP^Sc^, although they also show some specific structural nuances. Therefore, we are convinced that such recPrP^Sc^ constitute an excellent tool for future additional structural studies. It is noteworthy that cryoEM images of our samples (S7 Fig) as expected, showed fibrils that are very similar to those seen in brain-derived GPI-anchorless PrP^Sc^ samples. In summary, recPrP^Sc^ samples will be very useful in future structural studies based on the use of NMR.

## Supporting information

S1 Fig PK-resistant fragments are recovered in the pellet after centrifugation at 18.000 g. RecMoPrP^Sc^-950 was treated with 10 μg/ml PK at 37 °C for 30 minutes; the reaction was terminated by adding 2 mM Pefabloc. The sample was then centrifuged at 18.000g at 4 °C for 1 h. The pellet (P) was resuspended in a volume of 6M Gn/HCl equal to that of the supernatant (S). Protein in both supernatant and pellet fractions were precipitated with 85% methanol, and methanol pellets subjected to SDS-PAGE and immunobloted with antibody R1.
(TIFF)

S2 Fig ESI-TOF analysis of recMoPrP^Sc^-950 PK-resistant peptides. The PK digest was precipitated with methanol and the pellet dissolved in 6M Gn/HCl. The sample was then subjected to HPLC and the effluent fed to an ESI-TOF detector. The inset shows the total ion current output. The MS spectrum of the peak in at 18.9–19.6 minutes is shown. Deconvolution of the spectrum yielded a main component with an average mass of 9400.76 Da (MH+), corresponding to a peptide with the Mo PrP sequence M_153_-S_230_ (theoretical average mass: 9399.5 Da). AN Additional species with an MH+ value of +17 and likely corresponds to the same peptide with one of the three Met residues present in the sequence oxidized. A minor component with an MH+ value of 9531.61 Da might correspond to N_152_-S_230_ (theoretical average mass: 9513.6) with an oxidized methionine a component with M = 9514 Da.
(TIF)

S3 Fig Sequences of mouse and bank vole PrP. Secondary structure of PrP^C^, as determined by NMR studies (Mouse PDB 2L39; bank vole PDB 2K56): Lines placed on the PrP^C^ sequence indicate the location of β-sheet and α–helical regions.
(TIF)

S4 Fig Additional epitope mapping of PK-resistant peptides from recMoPrP^Sc^-950. RecMoPrP^Sc^-950 was treated with 10 μg/ml PK as described in the Materials and methods sections, and under the same conditions used for the experiments described in Fig 3. Digested samples were subjected to Tris/Tricine SDS-PAGE, electroblotted and probed with monoclonal antibody SAF84, which recognizes epitope 166–172. SAF84, as expected, recognized the ~16 and ~14.6 kDa bands corresponding to 86/98-230 and 117/119-230 fragments, and more weakly, ~13, ~12, and ~10.2 kDa bands putatively corresponding to ~117/119-230, ~135–230, and ~152/153-230. Essentially no bands were detected with smaller apparent MW values, since they do not contain the (166–172) epitope. Also, none of the doubly truncated fragments detected by 3F10, which do not include the epitope, were detected.
(TIF)

S5 Fig Histopathological and immunohistochemical analysis of brains from bank voles inoculated with recBVPrP^Sc^. Two brain areas were investigated: striatium and retrosplenial cortex. Spongiform lesion was studied by haematoxylin-eosin (H&E) staining. PrP^Sc^ aggregates/deposits were observed by IHC staining (antibody 6C2). Finally astroglia activation was determined by GFAP staining.
(TIF)

S6 Fig MALDI spectra of tryptic digests of bands 1 to 5 of the gel shown in Fig 5, corresponding to PK-treated recBVPrP^Sc^. The bands were excised and reduced, alkylated and digested in-gel with trypsin. Peptides were extracted, dried *in vacuo*, redissolved in 6M Gn/HCl and analyzed by MALDI-TOF [35].
(TIFF)

S7 Fig Cryo-electron microscopy of recMoPrP^Sc^. A PK-treated MoPrP^Sc^-17kDa sample was concentrated by centrifugation at 19,000 *g* for 1 hat 4°C (Sorvall ST 16R, Thermo Scientific). The resulting pellet was resuspended in 10 μl PBS (Fisher Bioreagents) and stored at -20 °C until microscopic examination. The sample was applied directly to a carbon-coated grid, R 2/2 Quantifoil^®^ (Quantifoil), and rapidly plunged into liquid ethane using a Vitrobot (Maastricht Instruments BV). Sample analysis was performed with a JEM 2200F (JEOL) transmission cryo-electron microscope, using an acceleration voltage of 200 KV and defocus ranging from 21.2 to 22.5 mm, determined accurately by using enhanced power spectra. Images were obtained with a 2k62k TM Ultrascan 1000 CCD camera (Gatan).
Images show short, untwisted fibrils, ~10 nm wide, apparently composed of two intertwined protofilaments, very similar to those of GPI-anchorless PrP^Sc^ [26]. Rosettes are glycogen present as a contaminant in the liver RNA extract used as part of the conversion mixture, as described before (Timmes et al. PLoS One. 2013 Jul 30;8(7):e71081. doi: 10.1371/journal.pone.0071081).
(TIF)
