# Supplementary figures and images for: Recombinant PrPSc shares structural features with brain-derived PrPSc: Insights from limited proteolysis
Source: PLoS Pathog. 2018 Jan 31;14(1):e1006797. doi: 10.1371/journal.ppat.1006797 (PMC5809102; doi:10.1371/journal.ppat.1006797)

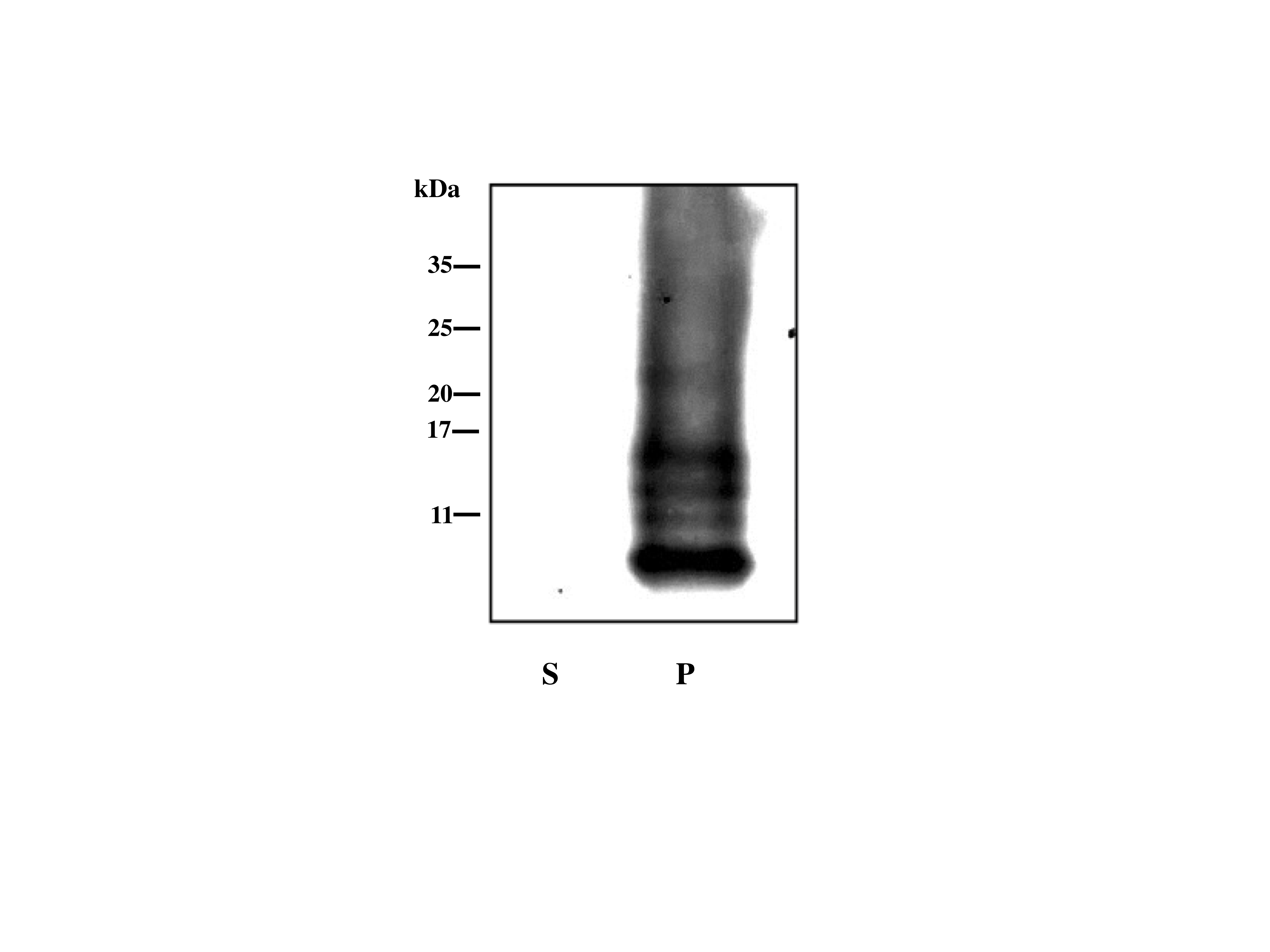

Supplement: S1 Fig — RecMoPrPSc-950 was treated with 10 μg/ml PK at 37 °C for 30 minutes; the reaction was terminated by adding 2 mM Pefabloc. The sample was then centrifuged at 18.000g at 4 °C for 1 h. The pellet (P) was resuspended in a volume of 6M Gn/HCl equal to that of the supernatant (S). Protein in both supernatant and pellet fractions were precipitated with 85% methanol, and methanol pellets subjected to SDS-PAGE and immunobloted with antibody R1. (TIFF) [file ppat.1006797.s001.tiff]

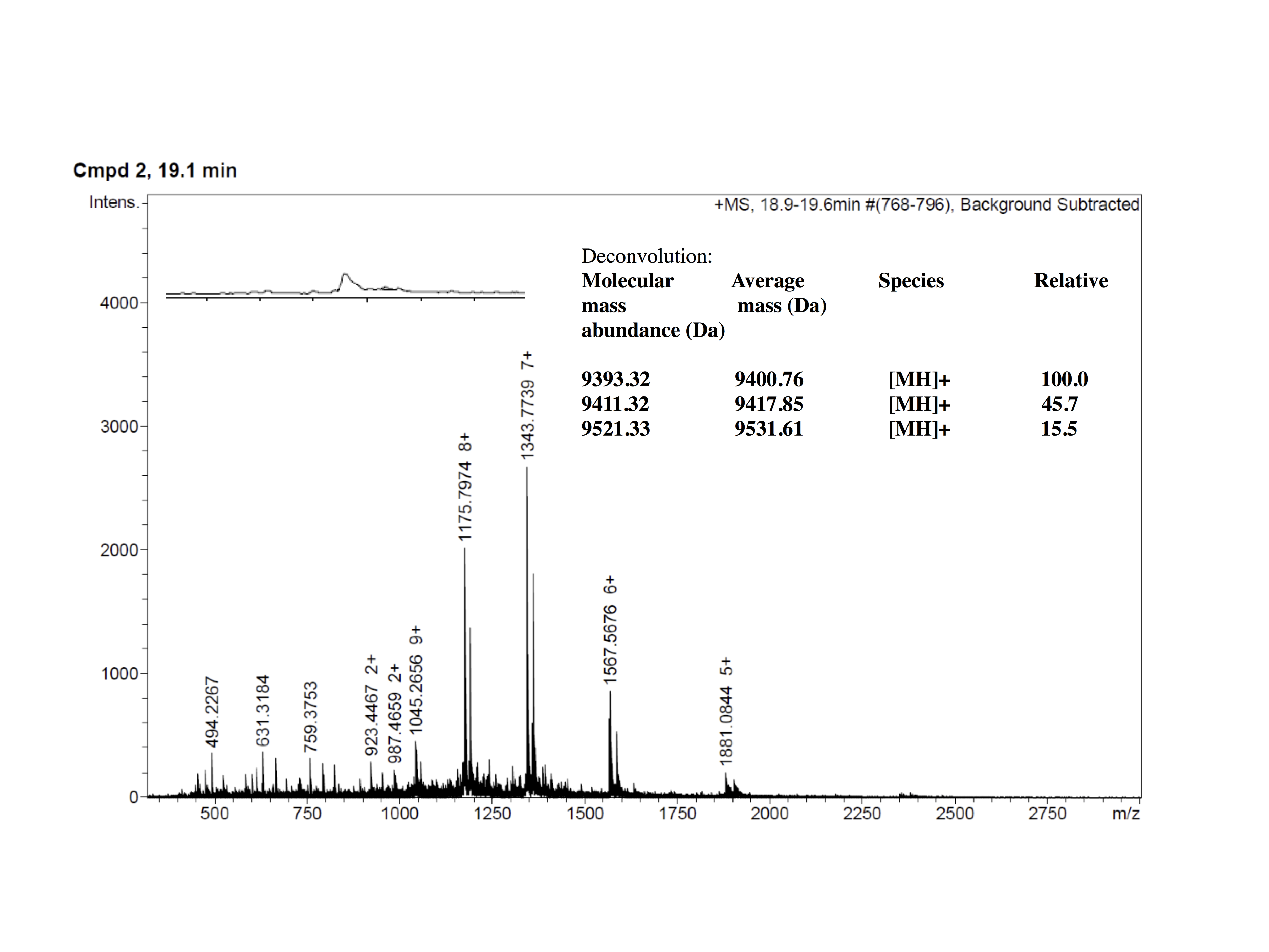

Supplement: S2 Fig — The PK digest was precipitated with methanol and the pellet dissolved in 6M Gn/HCl. The sample was then subjected to HPLC and the effluent fed to an ESI-TOF detector. The inset shows the total ion current output. The MS spectrum of the peak in at 18.9–19.6 minutes is shown. Deconvolution of the spectrum yielded a main component with an average mass of 9400.76 Da (MH+), corresponding to a peptide with the Mo PrP sequence M153-S230 (theoretical average mass: 9399.5 Da). AN Additional species with an MH+ value of +17 and likely corresponds to the same peptide with one of the three Met residues present in the sequence oxidized. A minor component with an MH+ value of 9531.61 Da might correspond to N152-S230 (theoretical average mass: 9513.6) with an oxidized methionine a component with M = 9514 Da. (TIF) [file ppat.1006797.s002.tif]

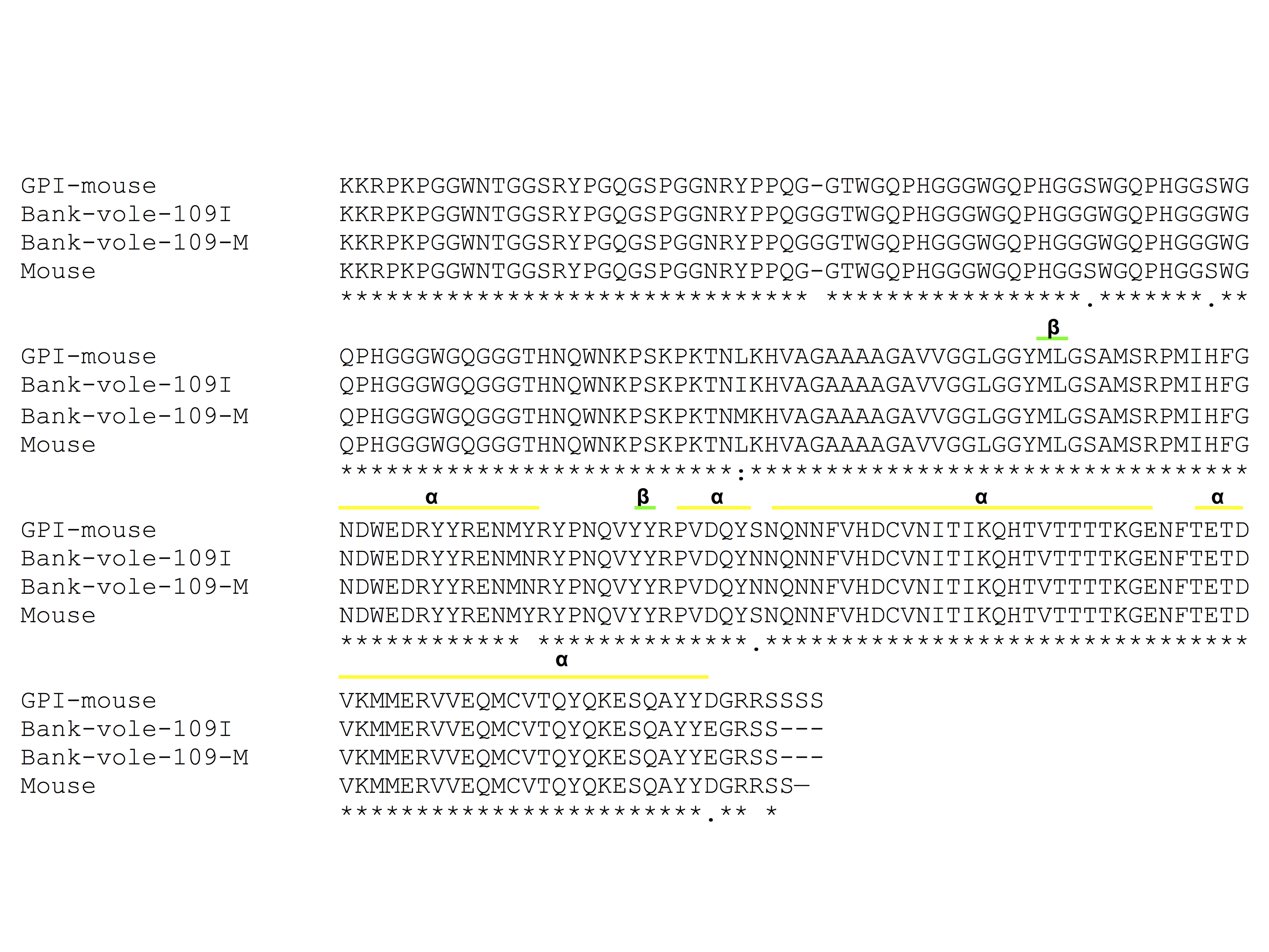

Supplement: S3 Fig — Secondary structure of PrPC, as determined by NMR studies (Mouse PDB 2L39; bank vole PDB 2K56): Lines placed on the PrPC sequence indicate the location of β-sheet and α–helical regions. (TIF) [file ppat.1006797.s003.tif]

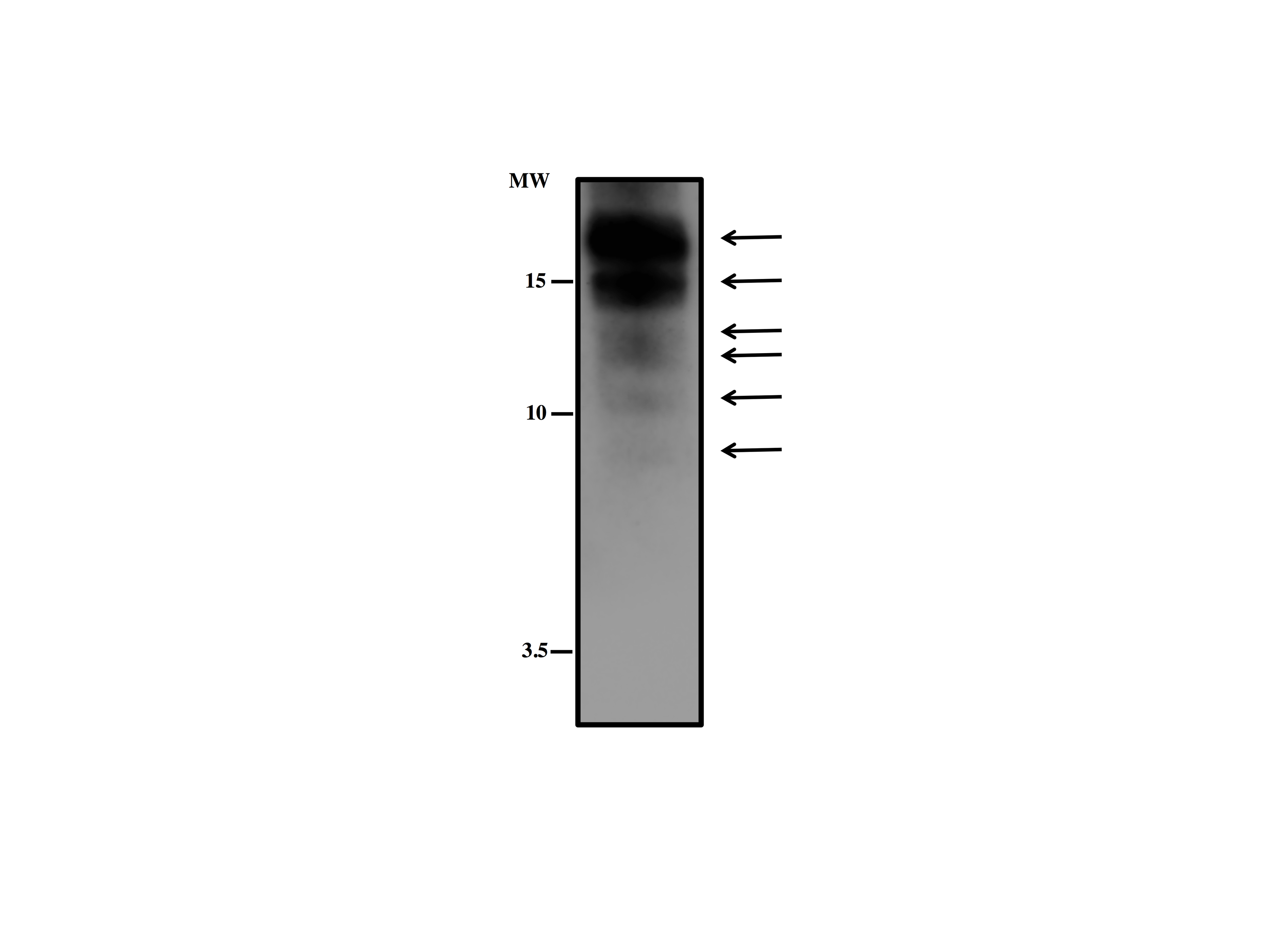

Supplement: S4 Fig — RecMoPrPSc-950 was treated with 10 μg/ml PK as described in the Materials and methods sections, and under the same conditions used for the experiments described in Fig 3. Digested samples were subjected to Tris/Tricine SDS-PAGE, electroblotted and probed with monoclonal antibody SAF84, which recognizes epitope 166–172. SAF84, as expected, recognized the ~16 and ~14.6 kDa bands corresponding to 86/98-230 and 117/119-230 fragments, and more weakly, ~13, ~12, and ~10.2 kDa bands putatively corresponding to ~117/119-230, ~135–230, and ~152/153-230. Essentially no bands were detected with smaller apparent MW values, since they do not contain the (166–172) epitope. Also, none of the doubly truncated fragments detected by 3F10, which do not include the epitope, were detected. (TIF) [file ppat.1006797.s004.tif]

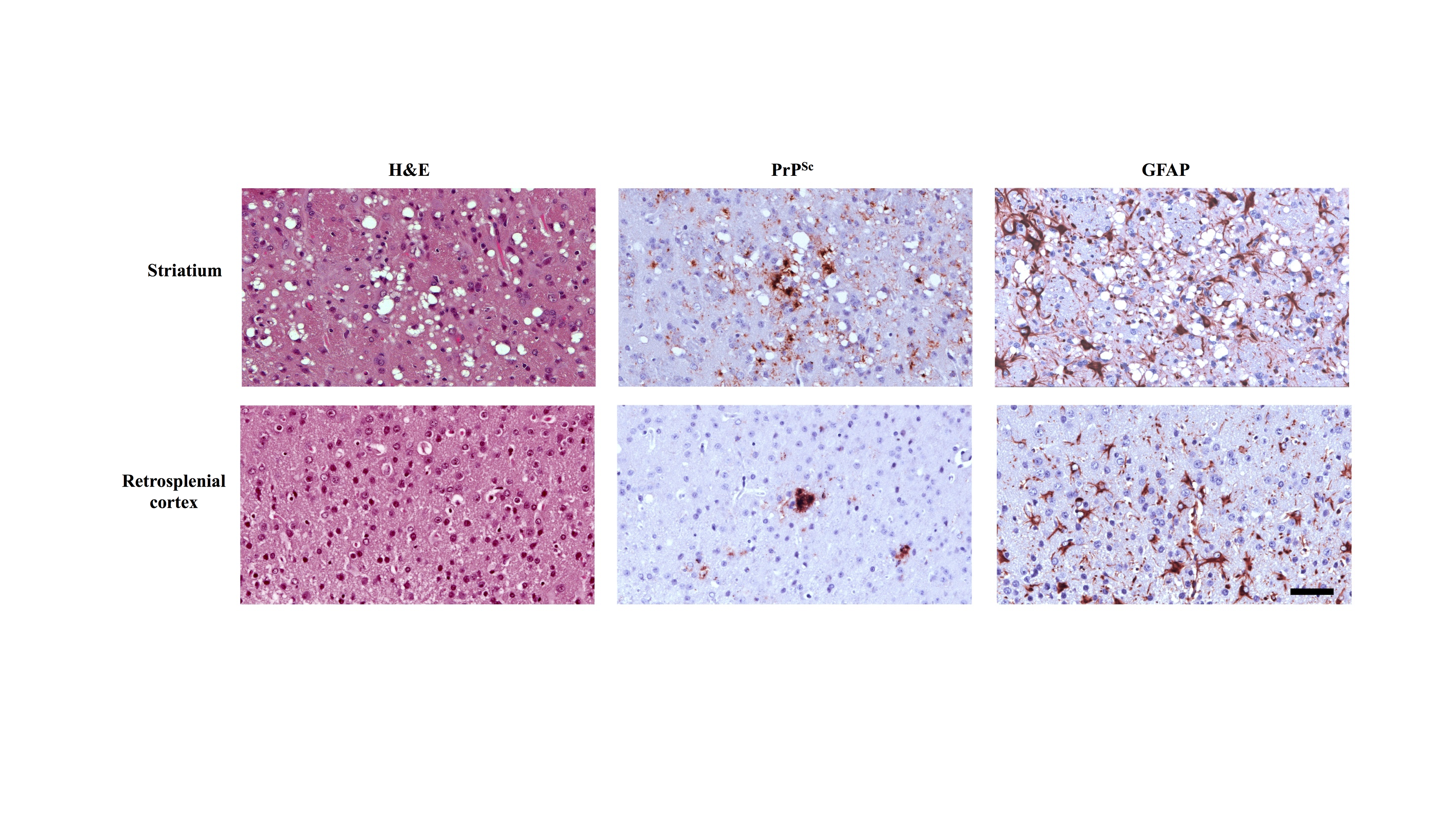

Supplement: S5 Fig — Two brain areas were investigated: striatium and retrosplenial cortex. Spongiform lesion was studied by haematoxylin-eosin (H&E) staining. PrPSc aggregates/deposits were observed by IHC staining (antibody 6C2). Finally astroglia activation was determined by GFAP staining. (TIF) [file ppat.1006797.s005.tif]

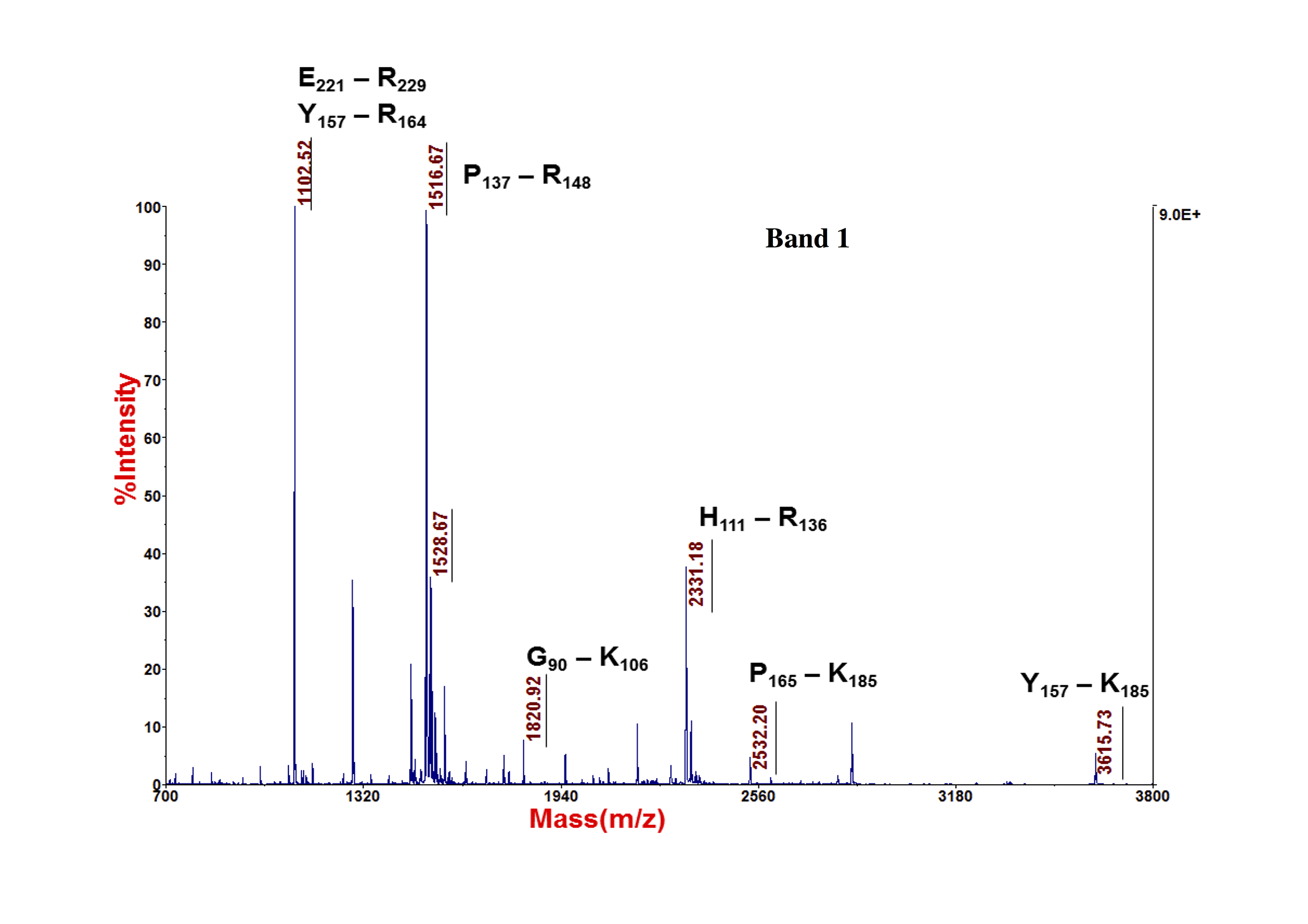

Supplement: S6 Fig — The bands were excised and reduced, alkylated and digested in-gel with trypsin. Peptides were extracted, dried in vacuo, redissolved in 6M Gn/HCl and analyzed by MALDI-TOF [35]. (TIFF) [file ppat.1006797.s006.tiff]

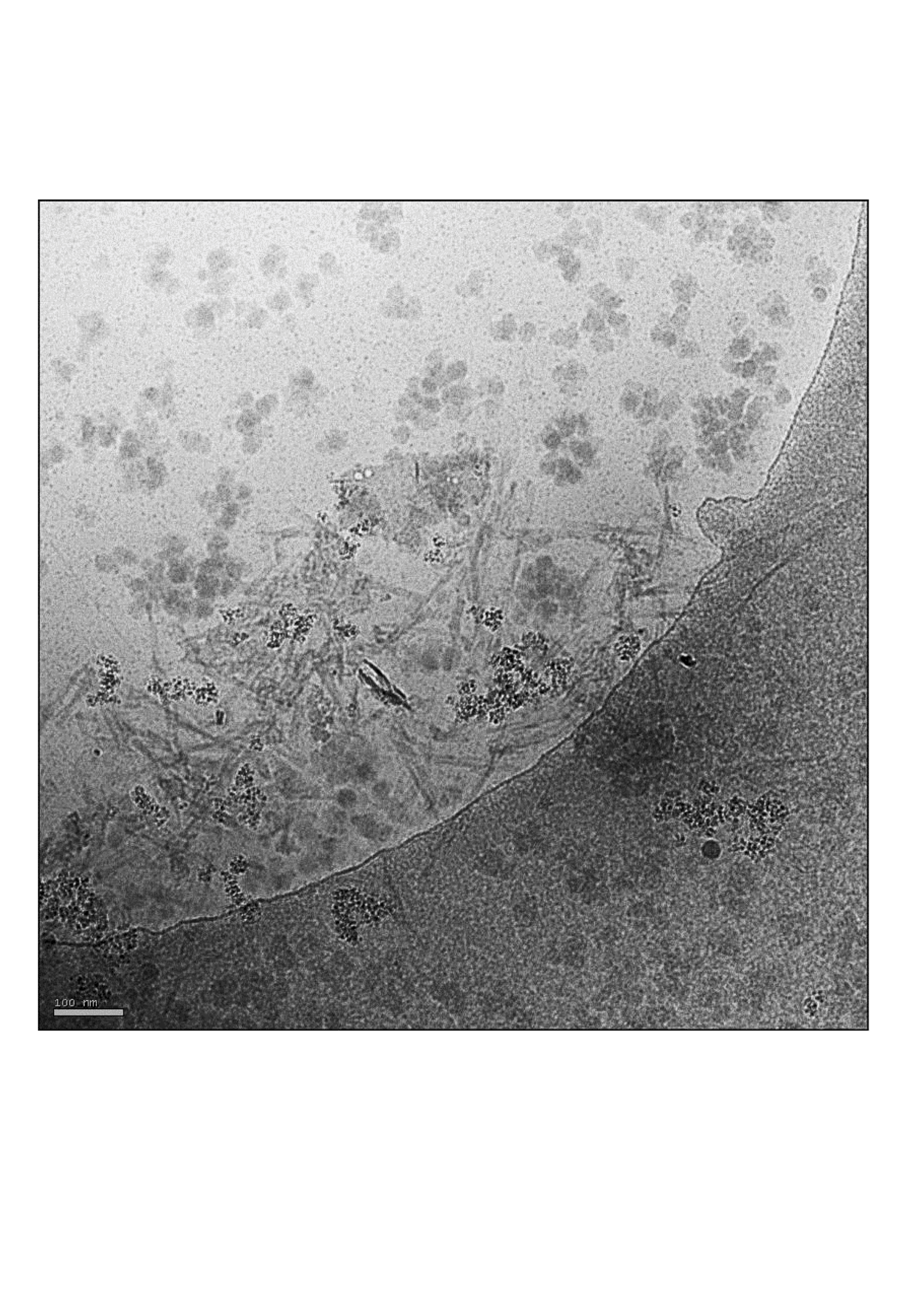

Supplement: S7 Fig — A PK-treated MoPrPSc-17kDa sample was concentrated by centrifugation at 19,000 g for 1 hat 4°C (Sorvall ST 16R, Thermo Scientific). The resulting pellet was resuspended in 10 μl PBS (Fisher Bioreagents) and stored at -20 °C until microscopic examination. The sample was applied directly to a carbon-coated grid, R 2/2 Quantifoil® (Quantifoil), and rapidly plunged into liquid ethane using a Vitrobot (Maastricht Instruments BV). Sample analysis was performed with a JEM 2200F (JEOL) transmission cryo-electron microscope, using an acceleration voltage of 200 KV and defocus ranging from 21.2 to 22.5 mm, determined accurately by using enhanced power spectra. Images were obtained with a 2k62k TM Ultrascan 1000 CCD camera (Gatan). Images show short, untwisted fibrils, ~10 nm wide, apparently composed of two intertwined protofilaments, very similar to those of GPI-anchorless PrPSc [26]. Rosettes are glycogen present as a contaminant in the liver RNA extract used as part of the conversion mixture, as described before (Timmes et al. PLoS One. 2013 Jul 30;8(7):e71081. doi: 10.1371/journal.pone.0071081). (TIF) [file ppat.1006797.s007.tif]
